# Mental health status of COVID-19 survivors: a cross sectional study

**DOI:** 10.1186/s12985-021-01729-3

**Published:** 2022-01-06

**Authors:** Munam Raza Jafri, Anna Zaheer, Sahar Fatima, Taiba Saleem, Atif Sohail

**Affiliations:** University Institute of Physical Therapy, The University of Lahore, Lahore, Pakistan

**Keywords:** COVID-19, Stress, Depression, Pandemic, Anxiety

## Abstract

**Background:**

Coronavirus disease-19 (COVID-19) is a communicable disease caused by a virus named severe acute respiratory syndrome coronavirus 2 (SARS-CoV-2) Pandemics are associated with the high level of mental stress. In many countries, general people reported the high level of depression, anxiety, psychological distress, post-traumatic stress disorder during recent a pandemic. This study aims to investigate the mental health status of people who survived through this alarming situation of COVID-19.

**Methods:**

In this study, seventy individuals (either gender) between the age of 18–60 years, who contracted COVID-19 previously and then recovered as indicated by negative PCR results, were included. Data was collected by using three tools: impact of event scale (IES-R), patient health questionnaire-9(PHQ-9) and corona anxiety scale (CAS). People with other systemic/mental disorders, ongoing malignancies, upper/lower motor disorders and inability to give consent were excluded from the study.

**Results:**

Mean age of participants was 26.29 + 11.79. All the 70 responders suffered from COVID-19. Among these 23 (32.9%) were asymptomatic and 47(67.1%) had common symptoms related to COVID-19 53 (75.7%) responders also had symptoms post-recovery. Most of the people who suffered COVID-19 had mild depression. Twenty-nine participants (41.4%) reported the highest impact of this traumatic event on their mental health. After suffering from COVID-19, 74.3% reported no anxiety as measured through corona anxiety scale (CAS).

**Conclusion:**

High level of post-traumatic stress was seen among participants who recovered from COVID-19, especially those patients who were symptomatic. Mild depression and anxiety were also noted among them.

## Background

COVID-19 is one of the main pathogens that chiefly affect the respiratory system of the human body. Earlier outbreaks of this virus include the Middle East respiratory syndrome (MERS-CoV) and the severe acute respiratory syndrome (SARS-CoV-2) which were formerly characterized as threat to public health [1]. Nevertheless, COVID-19 demonstrated some distinctive clinical features targeting respiratory tract symptoms like sneezing, sore throat and rhinorrhea [2]. Particularly dyspnea and fatigue were persistent in 87.4% of patients who had recovered from COVID-19, after negative PCR test [3].

In December 2019 five patients were admitted to the hospital having acute respiratory distress syndrome. 41 patients were hospitalized by January 2, 2020 and have been diagnosed with positive COVID-19 infection, around half of these patients had some underlying diseases which include hypertension, diabetes and cardiovascular diseases. The cumulative incidence of positive COVID-19 cases reached 5502 in China on January 29, 2020. And by January 30, 2020, almost 7734 positive cases have been identified in China and from several countries including Nepal, Sri Lanka, Japan, Republic of Korea, Singapore, Cambodia, United States of America, Canada, India, France, Germany, Finland, The Philippines, Thailand, Taiwan, and Malaysia [4]. In Pakistan, first case of COVID-19 was confirmed in February 2020. Considering the increasing number of confirmed cases in 2021 along with deficits in the healthcare delivery system, the viral disease spread just like wildfire across country.

After around an incubation period of 5 days, symptoms of COVID-19 start appearing which in few cases might lead to fatality. The time period between the onset of symptoms of COVID-19 and death ranges from 6 to 41 days. This time period depends upon many factors such as age and strength of patient’s immunity. Among older people, this period is relatively much shorter compared to people younger than 70 years of age. Cough, fever, fatigue are common occurring symptoms of COVID-19. While other symptoms include headache, hemoptysis, diarrhea and dyspnea etc. [5].

Pandemics are associated with the high level of mental stress. Fear, uncertainty, and stigma exist in any biological calamity, and they might operate as roadblocks to adequate mental therapy. Individuals are affected psychologically by the distress and uncertainty produced by the absence of proper management strategies for the COVID-19 pandemic. It has the potential to cause psychological disturbances [6]. Moreover, quarantine over an extended length of time might result in psychological stress reactions. Individuals who were confined to their homes tended to utilize social media to learn about the epidemic, exposing them to false information and unverified stories [7].

In many countries, general people reported the high level of depression, anxiety, psychological distress, post-traumatic stress disorder during a recent pandemic. Major risk factors related to mental disorders during COVID-19 pandemic are younger age, female gender, co-morbid diseases, other psychiatric disorders, and students by occupation, unemployment, more exposure to news or social media information concerning COVID-19 [8].

Emergence of COVID-19 outbreak relevant to Severe Acute Respiratory Syndrome COVID-19 2 (SARS-CoV-2) infection from China, a situation of severe psychological distress and socio-economic crisis speedily occurred worldwide. This psychological distress includes anxiety, frustration, stress and depression led to a vague situations which emerged progressively during this outbreak [9]. Many tools are available to measure different aspects of mental status or psychological well-being, among which impact of event scale (IES-R), patient health questionnaire-9(PHQ-9) and corona anxiety scale (CAS) are used in this study to measure stress, depression and anxiety.

Being a low-middle income country, Pakistan encountered lots of health care challenges in the last many decades. The country had to endure many devastating effects of epidemics on people in terms of economic loss, morbidity and mortality [10]. Economical security, fear, mental health uncertainty are a few devastating impacts of the COVID-19 pandemic and lockdown resulting from it.

Pakistan, being a developing country with limited resources lack proper surveillance systems, trained human resources and laboratory networks. Within limited resources Pakistan has made strategies sufficient to prepare for dealing this global breakout [11]. However, to prevent various negative consequences, mental and psychological strategies are required. For this purpose, this study aims to investigate mental health status of people who survived this alarming situation of the COVID-19.

## Methods

A cross-sectional study was conducted on COVID-19 survivors from the city of Lahore. People (either gender) between the age of 18–60 years, who contracted COVID-19 previously and then recovered as indicated by negative PCR results, were included in the study. People with other systemic/mental disorders, ongoing malignancies, upper/lower motor disorders, inability to give consent and people who were having any kind of therapies for their psychological well-being were excluded from the study. Participants were recruited on the basis of informed consent and ethical approval was taken from the institution reviewer committee of The University of Lahore, Pakistan. The Sample size was seventy as calculated by epitool [12]. The Convenient sampling technique was applied. Data was collected through questionnaire consisting of demographic data and three tools: impact of event scale (IES-R), patient health questionnaire-9(PHQ-9) and corona anxiety scale (CAS). IES-R measures distress related to any traumatic event. PHQ-9 is a multipurpose tool for measuring and diagnosing the level of depression. CAS is a self-report screening tool for measuring mental health associated with the COVID-19 survivors having dysfunctional anxiety.

### Data analysis

Data was entered and statistically analyzed using SPSS version 22.0. P value ≤ 0.05 was considered as significant. Results were represented as frequency and percentages.

## Results

Seventy COVID-19 survivors participated in this study among which 28 were males and 42 were females. Participants aged between 18 and 60 years (mean 26.29 + 11.79) were included. All the responders suffered from COVID-19. Among these 23(32.9%) were asymptomatic and 47(67.1%) had common symptoms related to COVID-19. Sixty-five (92.9%) participants were quarantined at their homes while other 5 (7.1%) were admitted in different hospitals of Lahore after they were diagnosed with COVID-19. However, none of the participants were ventilated in any point of their treatment. The study showed that 53(75.7%) responders also had symptoms post recovery.

**Fig. 1:**
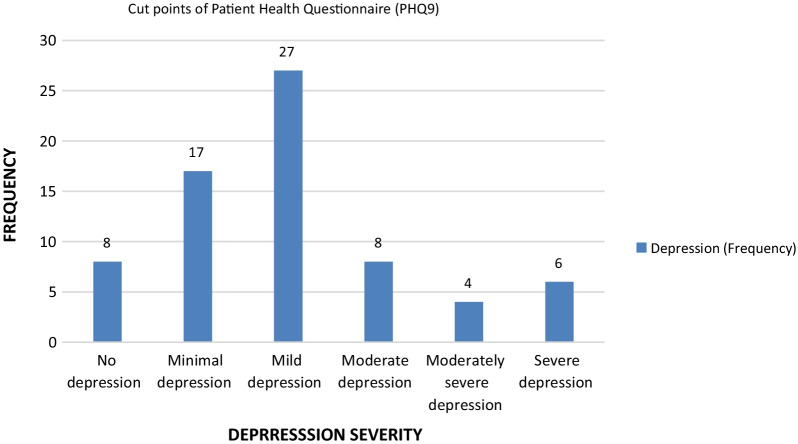
Descriptive statistics of depression severity (Patient Health Questionnaire-9)

Depression: Patient health questionnaire (PHQ-9) was used to measure levels of depression. Among 70 participants only 8 (11.4%) were not suffering from depression-related symptoms. Twenty-seven (38.5%) participants had mild depression. Remaining 18 (25.7%) participants who suffered COVID-19 had moderate to severe depression (Fig. 1). 19(40.4%), 6(12.7%), 2(4.3%) and 6(12.7%) symptomatic patients had mild depression, moderate depression, moderately severe depression and severe depression, respectively. Few asymptomatic patients suffered mild to moderate depression but no one from this group suffered severe depression.

Post-traumatic stress disorder: After recovery from COVID-19, 23 (32.9%) of all 70 responders showed no impact of COVID-19 on their mental status as measured through impact of event scale (IES-R). However, 47(67.1%) survivors showed post-traumatic stress. Twenty-nine (41.4%) reported highest impact of this traumatic event on their mental health. Other showed partial post-traumatic stress disorder (Table 1). 25(53%) symptomatic COVID-19 patients suffered high impact of event (PTSD) but only 4(17.3%) asymptomatic patients suffered from this level of stress.

**Table 1:** Descriptive statistics of impact of event scale (IES-R) scores

	Frequency	Percent (%)
No impact	23	32.9
Partial (PTSD)	11	15.7
Probable diagnosis (PTSD)	7	10.0
High impact of event (PTSD)	29	41.4
TOTAL	70	100.0

*PTSD* post-traumatic stress disorder

Anxiety: After suffering from COVID-19, 18 (25.7%) survivors showed corona related anxiety while 52 (74.3%) reported no anxiety as measured through corona anxiety scale (CAS) (Table 2). Among symptomatic patients 14(29.7%) suffered corona related anxiety. However, only 4(8.5%) asymptomatic patients were going through corona related anxiety.

**Table 2:** Frequency of COVID-19 related anxiety among participants through CAS (Corona Anxiety Scale)

	Frequency	Percent (%)
Corona related anxiety	18	25.7
No anxiety	52	74.3
Total	70	100.0

## Discussion

The average mean age in this study was 26 years (standard deviation = 11.791 years). In various other studies the range of age was 30–70 years. In a study conducted by Michiko Ueda, et al., studied the status of mental health of general population during COVID-19 pandemic and it included 2000 respondents. They concluded that not only health consequences of COVID-19 pandemic but also financial vulnerability during pandemic have affected mental health status of general population especially working age population [13].

Result of this study showed that majority of the COVID-19 survivor population has symptoms of mild depression. In another study of Chaomin Wu et al. concluded that only 10% of the COVID-19 survivor population, especially females, has symptoms of depression and anxiety due to contagious nature of infection, post discharge symptoms and fear of recurrence [14]. The current study showed quite opposite results in terms of depression. Most of the COVID-19 survivors have mild depression and high impact of event (post-traumatic stress disorder). On the other hand, there is significantly lesser number of participants who were suffering from corona related anxiety.

A study conducted by Yu.Fen Ma et al. on prevalence of depression and its association of quality of life in clinical stable patient with COVID-19. He demonstrated that the prevalence of depression was high and appropriate treatment and regular screening of depression was suggested. The result of this study is coincided with the current study because in times of pandemic there were circumstances of panic everywhere and many people around the world lost jobs. This pandemic hit worse on daily wagers. These are the factor here that adds up in anxiety and depression to the population especially COVID-19 survivors [15].

This study demonstrates high level of stress (post-traumatic stress). It might be because of the reaction of people around them, quarantine condition and the news they receive from electronic and social media regarding on-going pandemic situation which causes distress among them. Altefeh Zandifar et al., also described prevalence and severity of depression, anxiety, stress and perceived stress in hospitalized patient with COVID-19. The study demonstrated that all the participants had severe anxiety and almost 98% of participants had some degree of depression and stress [16].

Regarding correlation between COVID-19 type (symptomatic or asymptomatic), the group with symptomatic COVID-19 has high levels of depression, anxiety and stress as compared with asymptomatic COVID-19 patients. As having the symptoms of this contagious viral disease may lead to, anger, guilt and uncertainty. A person have to be isolated as a stigma prevails among family and friends. Therefore, they feel more psychological distressed. These findings are consistent with the results of a study which showed that psychological distress was more prevalent among patients with symptomatic COVID-19 as compared to asymptomatic COVID-19 patients [17].

Even after the traumatic event ends, people may have disturbing intense feelings and thoughts related to experience that had. People may recall the stressful experience through flashbacks and nightmares. They may detach themselves from things that may remind themselves of that traumatic event. According to this study, majority of the participants had stress even after having negative findings in their COVID-19 test results. This is also shown in results of the study conducted by Mengyuan Dong et al. The news about the pandemic made general public to feel panic and anxiety. The use of mobile phone and instant messaging applications exacerbate the issue by spreading news faster. This psychological disorder was named as “headline stress disorder [18].

Moreover, results showed that psychological distress was more prevalent among people with younger age and female gender. Many females suffer increased stress as they have more responsibilities towards family. Studies showed poor sleep quality, loneliness were directly related to anxiety in young people. People above 35 years of age were highly resilient which is inversely proportional to their anxiety level [19]. These results are consistent with a study which revealed that the factors which had significant greater odds of having mild to extreme levels of stress, anxiety and depression are: age equal and younger than 25 years, female gender and having COVID-19 symptoms within last 14 days [20]. Moreover, people having comorbidities showed more psychological distress than people with asymptomatic COVID-19 and no co-morbidities.

According to this study psychological issues should be addressed after recovery from COVID-19 because in long term mental health is negatively affected by mild level of anxiety, depression and high level of post-traumatic stress. Demographic differences were not included in the study and there was lack of generalization of results because of small sample size.

## Conclusion

High level of post-traumatic stress was seen among participants who recovered from COVID-19, especially those patients who were young, had comorbidities, symptomatic COVID-19 and female gender. Mild depression and anxiety were also noted among them. Moreover, it can be concluded that there is a need for more efficient psychological assessment which can help in designing appropriate psychological interventional strategies.

## Data Availability

All data generated or analyzed during this study are included in this published article [and its supplementary information files].

## Abbreviations

COVID-19: Coronavirus Disease 2019
IES-R: Impact of event scale
PHQ-9: Patient health questionnaire-9
CAS: Corona anxiety scale
MERS-CoV: Middle East respiratory syndrome coronavirus
SARS-CoV-2: Severe acute respiratory syndrome coronavirus 2
